# Novel flavonoid* C-8* hydroxylase from *Rhodotorula glutinis*: identification, characterization and substrate scope

**DOI:** 10.1186/s12934-022-01899-x

**Published:** 2022-08-29

**Authors:** Kinga Dulak, Sandra Sordon, Agata Matera, Bartosz Kozak, Ewa Huszcza, Jarosław Popłoński

**Affiliations:** 1 Department of Food Chemistry and Biocatalysis, Wroclaw University of Environmental and Life Sciences, Wrocław, Poland; 2 Department of Genetics, Plant Breeding and Seed Production, Wroclaw University of Environmental and Life Sciences, Wrocław, Poland

**Keywords:** Monooxygenase, Flavonoids, Yeasts, *Rhodotorula glutinis*, Synthetic biology, *Ortho*-hydroxylation, FMO, Biotransformation

## Abstract

**Background:**

The regioselective hydroxylation of phenolic compounds, especially flavonoids, is still a bottleneck of classical organic chemistry that could be solved using enzymes with high activity and specificity. Yeast *Rhodotorula glutinis* KCh735 in known to catalyze the C-8 hydroxylation of flavones and flavanones. The enzyme F8H (flavonoid C8-hydroxylase) is involved in the reaction, but the specific gene has not yet been identified. In this work, we present identification, heterologous expression and characterization of the first F8H *ortho*-hydroxylase from yeast.

**Results:**

Differential transcriptome analysis and homology to bacterial monooxygenases, including also a FAD-dependent motif and a GD motif characteristic for flavin-dependent monooxygenases, provided a set of coding sequences among which RgF8H was identified. Phylogenetic analysis suggests that RgF8H is a member of the flavin monooxygenase group active on flavonoid substrates. Analysis of recombinant protein showed that the enzyme catalyzes the C8-hydroxylation of naringenin, hesperetin, eriodyctiol, pinocembrin, apigenin, luteolin, chrysin, diosmetin and 7,4ʹ-dihydroxyflavone. The presence of the C7-OH group is necessary for enzymatic activity indicating *ortho*-hydroxylation mechanism. The enzyme requires the NADPH coenzyme for regeneration prosthetic group, displays very low hydroxyperoxyflavin decupling rate, and addition of FAD significantly increases its activity.

**Conclusions:**

This study presents identification of the first yeast hydroxylase responsible for regioselective C8-hydroxylation of flavonoids (F8H). The enzyme was biochemically characterized and applied in in vitro cascade with *Bacillus megaterium* glucose dehydrogenase reactions. High in vivo activity in *Escherichia coli* enable further synthetic biology application towards production of rare highly antioxidant compounds.

**Supplementary Information:**

The online version contains supplementary material available at 10.1186/s12934-022-01899-x.

## Introduction

Flavin-dependent monooxygenases (FMOs) are among the most important microsomal proteins involved in many biological processes [1]. They play a key role in the catabolism of natural compounds, support the biosynthesis of hormones, vitamins and antibiotics. They are also involved in defense strategies [2] and metabolism of non-nutritional compounds, drugs, and xenobiotics [3].

The mechanism of action of FMO is clearly different from that of other monooxygenases. In contrast to cytochrome P-450-dependent enzymes, FMOs do not require an additional reductase to function [4].

Selective hydroxylation of aromatic compounds is still a challenging chemical reaction in synthetic chemistry and has been gaining increasing interest in recent years, especially due to the use of hydroxylated aromatic compounds in the pharmaceutical industry [5]. The use of isolated enzymes or whole cells for biocatalytic oxygen transfer is an environmental friendly, inexpensive and efficient way of targeted hydroxylation [2, 6–10].

The health-promoting activity of flavonoid compounds is mainly related to their antioxidant capacity. Flavonoids with C7–C8 catechol moiety show much higher antioxidant activity compared to C7-OH analogues [11]. The presence of catechol moiety is responsible for efficient radical scavenging [12], inhibition of lipid peroxidation [13], and complexation of metal ions [14]. The ability to complex transition metals is an important factor influencing the biological activity of flavonoids. It affects some oxidative processes, including reactions occurring as a result of radical stress. A special case is the inhibition of low-density lipoprotein oxidation and destructive oxidative processes involving nucleic acids. It also plays an important role in affecting capillaries and inhibiting the spread of inflammation [15]. It also engages in the induction of detoxifying phase II enzymes such as heme oxygenase-1 (HO-1), c-glutamyl cysteine ligase (c-GCL), and NADPH quinone oxidoreductase 1 (NQO1) [16]. As an example, butein (3-hydroxy-isoliquiritigenin) had better neuroprotective effects against glutamate-induced oxidative stress in HT22 cells than isoliquiritigenin. Hydroxylation of flavonoids, resulting in the formation of catechol groups, may facilitate further methylation of one of the vicinal hydroxyl groups [17]. Furthermore, the literature data indicate that catechol hydroxyl moieties in combination with free carboxylic acid are essential for the neuroprotective effects of carnosic acid [18]. However, the hydroxylation of flavonoids has not been widely studied to date. Recent literature reports give examples of hydroxylation at C-3ʹ, and C-6 positions mediated by enzymes belonging to the cytochrome P-450 monooxygenase group, requiring interaction with at least one oxidoreductase [8, 19–23]. A few examples of flavonoid hydroxylation at the C-8 position have been also described [21, 24].

The red yeast from the genus *Rhodotorula* was identified as microorganisms capable of efficient hydroxylation of naringenin. *Rhodotorula marina* AM77 transforms naringenin into a mixture of polyhydroxylated products: carthamidin (C-6) and isocarthamidin (C-8) [25]. The obtained mixture of C-8 and C-6 hydroxynaringenin shows much higher antioxidant activity than naringenin [26]. Broad screening on red yeasts performed by Sordon and co-workers indicated that also *Rhodotorula glutinis* KCh735 was capable of transformation of naringenin. The selected strain additionally catalyzed regioselective *ortho*-hydroxylation of other natural flavonoids belonging to the flavanones and flavones groups [27].

In this study, F8H from *R. glutinis* KCh735 responsible for naringenin hydroxylation was identified. The transcriptomes were analyzed on the basis of specific induction. Phylogenetic analysis suggests that RgF8H is classified as a novel flavoprotein monooxygenase. Heterologous expression of RgF8H in *Escherichia coli* cells showed NADPH-dependent activity towards flavonoids. This work presents the first cloning and identification of F8Hs responsible for *ortho*-hydroxylation from yeast.

## Experimental

### Chemicals

Naringenin, hesperetin, 6-hydroxyflavanone, 7-hydroxyflavanone, chrysin, 3-hydroflavone, morin, epicatechin, daidzein, resveratrol, lysozyme, and BSA (bovine serum albumin) were purchased from Sigma-Aldrich Chemical Co. (St. Louis, MO, USA). 2ʹ-hydroxyflavanone, 3ʹ-hydroxyflavanone, and 4ʹ-hydroxyflavanone were purchased from Alfa-Aesar (Thermo Fisher, Karlsruhe, Germany), and the other substrates were purchased from Carbosynth (Berkshire, UK). The structures of all substrates used in this work are summarized in Additional file 1: Table S1. All chemicals and medium (peptone from animal tissue, tryptone, yeast extract, glucose) compounds were purchased from Sigma-Aldrich (St Louis, USA), or Carbosynth (Compton, Berkshire, U.K.). Nicotinamide β-adenine dinucleotide phosphate (oxidized sodium salt hydrate), X-gal (5-bromo-4-chloro-3-indolyl β-d-galactopyranoside), and antibiotics were purchased from Cayman Chemical Company (Ann Arbor, Michigan, USA). Nicotinamide adenine dinucleotide and nicotinamide adenine dinucleotide phosphate were purchased from Merck Millipore (Burlington, Massachusetts, USA). The UPLC and LC–MS grade solvents used in this study were purchased from Biocorp (Poland). The yeast strain used in this study was *Rhodotorula glutinis* KCh735 that is deposited in the Microorganism Collection of the Department of Food Chemistry and Biocatalysis of Wroclaw University of Environmental and Life Sciences [27].

### Induction of C-8 hydroxylases

To determine whether the enzyme responsible for hydroxylation of flavonoids at C-8 in *R. glutinis* KCh735 is produced constitutively or being induced, 12 different flavonoids were tested. Yeast cultures were cultivated under aerobic conditions in 100 mL Erlenmeyer flasks with 30 mL of Sabouraud medium (glucose 3%, peptone 1%; m/m) at room temperature (22–23 ℃) and 130 rpm with a 5% inoculum overnight. After 24 h, cultures were induced to a total final flavonoid concentration of 0.05 µM, and added to the reaction mixture as a methanol stock solution (0.1 mL). The control reaction was carried out without the addition of the inducing agent (methanol only). The compounds used in the experiments are listed in Additional file 1: Table S2. Twelve hours after induction, 7.5 mg cycloheximide was added to each flask and 15 min later 5 mg of naringenin as a stock solution in DMSO (0.1 mL). The reaction was run overnight and then 15 mL of ethyl acetate was added to extract the reaction products. Cultures were shaken, centrifuged for 10 min at 4000 rpm and 5 mL of the organic fraction was collected, evaporated, and resuspended in 1 mL of methanol for analysis by UPLC–DAD. Three flavonoids, chrysin, naringenin, and apigenin, were selected for further studies. The effect of different concentrations of inducer (1–100 µM) on hydroxylase activity was analyzed following the same procedure.

### Bioinformatics analyses

The transcriptomes of two *R. glutinis* KCh735 strains were subjected to high-throughput sequencing by synthesis (SBS). Cultures of *R. glutinis* KCh735 were grown in Sabouraud medium and 0.1 mM naringenin was used to induce one of the cultures. Total RNA was isolated and purified from the cell pellet using the Monarch Total RNA Miniprep Kit (BioLabs) according to the manufacturer’s instructions. Total RNA was quantified using a spectrophotometer (Eppendorf BioSepctrometer Kinetic). The standard TruSeq mRNA strand preparation protocol was used for library preparation. Sequencing was performed on an Illumina hiseq 2500 using 150 PE reads. Library preparation and sequencing were performed according to the Macrogen service. The raw data obtained were used in a standard bioinformatics pipeline. In the first step, the raw data were cleaned by removing low-quality reads (Phred score below 20) and adaptor sequences. These steps were performed using trimmomatic software [28], and the quality of raw and clean reads was assessed using fastQC and MultiQC software [29, 30]. The cleaned reads obtained were used for de novo transcriptome reconstruction using Trinity software [31]. The software was run with standard parameters for band libraries. The obtained transcriptomes were evaluated by counting full-length Trinity transcripts and counting Nx and ExN50 statistics (GitHub—trinityrnaseq/trinityrnaseq: Trinity RNA-Seq de novo transcriptome assembly). In the next step, the obtained transcripts were annotated by blasting to a reference RNA database downloaded from GenBank (National Center for Biotechnology Information (nih.gov)). An alignment-based RSEM method was used to count transcriptomes [32]. Purified reads were mapped to the resulting transcriptome using Bowite 2. Then, the abundance of all transcripts in each sample was estimated using the RSEM algorithm. Finally, the array of counts was analyzed with the edgeR package [33], using the statistical software R to find transcripts with different expression levels between the analyzed samples.

### Identification of RgF8H

The transcripts with the greatest difference in expression of individual transcripts between induced and uninduced samples were screened for FAD-binding sequence, and homology to the hydroxylase sequence from *Herbaspirillum seropedicae* (*fdeE*) [34] using the nucleotide–nucleotide blast program (blastn) (National Center for Biotechnology Information (nih.gov)). Protein sequences were aligned using Clustal Omega software (https://www.ebi.ac.uk/Tools/msa/clustalo). The phylogenetic tree was constructed using iTOL software (https://itol.embl.de/). GenBank IDs of the used protein sequences (amino acids) are shown in Additional file 1: Table S3.

### Bacterial strains and plasmids

**Fig. 1:**
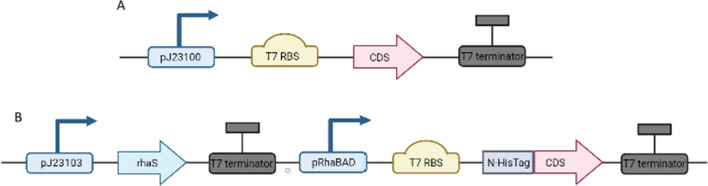
Schematic map of **A** constitutive and **B** induced expression cassette

The Standard European Vector Architecture (SEVA) vector series [35] were used for the expression of all selected yeast genes, that were codon-optimized and ordered from a commercial supplier (Doulix). *Escherichia coli* strain DH5α (NEB) was used for cloning, and BL21 (DE3) (NEB) was used as the expression host. Liquid cultures in LB medium (1% tryptone, 0.5% yeast extract, 0.5% NaCl; m/m) were incubated overnight at 37 ℃ and 120 rpm in 300 mL Erlenmeyer flasks. *E. coli* cells containing each plasmid (Additional file 1: Table S4) were selected by adding the antibiotic—kanamycin to the medium at a final concentration of 30 µg/mL and by Blue-White screening [36]. A summary of all cloning details including all *R. glutinis* sequences, primer sequences, corresponding vectors, and strains in which they were used are presented in Additional file 1: Table S4. The assembly of the vectors follows the Golden Standard modular cloning (GS MoClo) assembly procedure (data unpublished). Plasmids with potential hydroxylase from the *R. glutinis* coding sequence flanked by BsaI restriction sites and the empty vector pRhaBAD_12 containing the suitable promoter, transcription factor, and terminator sequences flanked by BbsI restriction sites were purchased from Gen Art. All *R. glutinis* genes were excised by the restriction enzyme BsaI-HFv2 and inserted together with pJ23100, T7 RBS, and the T7 terminator into pSEVA23g19g1 for in vivo studies. The plasmid harbouring the pRhaBAD_12 expression cassette site, with constitutively produced RhaS transcription regulator was digested with the restriction enzyme BbsI and inserted into pSEVA23g19g1. To verify protein overexpression, the constructed backbone vector pRhaBAD_12 was used, into which the T7 RBS and the N-6× histidine fusion tag sequences digested by BsaI-HFv2 were inserted (Fig. 1). All constructs were validated by sequencing (Macrogen Europe). All constructed plasmids are available upon request.

### Protein expression and purification

For recombinant protein purification, a pre-culture of 30 mL LB medium containing 30 µg/mL kanamycin was inoculated from glycerol stock (transformed *E. coli* strains with the description of their use for protein overexpression analysis are summarised in Additional file 1: Table S4) and shaken overnight at 37 °C. Subsequently, 100 mL of liquid culture containing the antibiotic was inoculated with 10% (v/v) of the pre-culture and shaken at 120 rpm at 37 °C. When OD_600_ reached 0.7, the culture was induced by adding 25 mM l-rhamnose and shaking overnight at 30 °C. The cultured cells were pooled and harvested by centrifugation at 14,000×*g* for 20 min at 4 °C. The pellets were resuspended in 5 mL Binding Buffer (20 mM sodium phosphate, pH 7.4, 500 mM NaCl, 20 mM imidazole) in which they were treated with 300 µg of lysozyme per 1 mL of suspension. Cells were disrupted by ultrasound for 5 min (pulse 5 s, pause 5 s, amplitude 85%, 130 W, 20 kHz) in the ice bath, centrifuged at 14,000×*g* for 20 min at 4 °C. The supernatant was applied to a 5 mL IMAC column (His-TrapTM, GE Healthcare). The His-tagged recombinant protein was eluted in Elution Buffer (20 mM sodium phosphate, pH 7.4, 500 mM NaCl, 500 mM imidazole), fractions were combined, directly used for enzyme assays, or stored at − 20 °C until use. Protein purification was evaluated via 4–20% SDS-PAGE electrophoresis, proteins were stained by Coomassie Brilliant Blue (Cepham Life Sciences, Inc). Precision Plus Protein Dual Color Standards (BIO-RAD) was used as a size marker. Protein concentrations were evaluated using the Bradford assay using BSA as a reference for calibration [37].

### Standard in vivo and in vitro activity assay

The standard in vivo activity test was performed as follows. *E. coli* cells expressing putative recombinant hydroxylase were grown aerobically at 37 °C in a 600 µL LB medium containing 30 µg/L of kanamycin. Substrates were added to a concentration of 0.01 mM as a DMSO stock solution (1 mM) and shaken overnight at 30 °C, 120 rpm. In the case of inducible expression 10 mM l-rhamnose was added after a culture reached the optimal density at OD_600_ = 0.7. Reactions were stopped and extracted with 200 µL of ethyl acetate, vortexed, centrifuged (14,000×*g*, 2 min) and 50 µL of a resulting top organic layer was transferred to 450 µL of methanol for direct analysis by UPLC–DAD.

A standard in vitro assay test was performed as follows. Enzymatic reactions were carried out (0.01 mM naringenin, 0.1 mM NADPH, 100 μL of crude or 20 µL of purified protein extract (1.5 mg/mL) in 25 mM sodium phosphate buffer (pH 7.5)) at 30 °C, 800 rpm for 1 h. Reactions were stopped with adding an equal volume of methanol, vortexed, centrifuged (14,000×*g*, 2 min), evaporated, dissolved in 1 mL of methanol, and 200 µL of a resulting alcoholic solution was direct analysis by UPLC–DAD.

### Biochemical characterization

In order to determine the optimal parameters for the enzymatic reaction catalyzed by RgF8H, a series of reactions were performed. The reactions were carried out at different temperatures (10–50 ℃), at different pH values ranging from 4.0–5.5 (CH_3_COOH–CH_3_COONa buffer), 6.0–8.0 (NaH_2_PO_4_–Na_2_HPO_4_ buffer), 8.0–9.0 (Tris–HCl buffer) and 9.0–10.0 (glycine buffer). The effect of different ionic strength (0–1500 mM), buffer molarity (5–100 mM), and co-solvent addition (0–30%) on RgF8H activity were also investigated using 25 mM phosphate buffer. Timely collected samples (100 µL) were mixed with 100 µL MeOH to stop the reaction and analyzed using a plate reader (BioTEK, SYNERGY H1) based on the decrease of NADPH concentration. The stability of the enzyme was determined by the ThermoFAD method [38]. Samples contained 10 µL (1.5 mg/mL) of purified RgF8H and 15 µL of 50 mM buffers with a pH range of 4.0–10.0. To test the effect of glycerol on enzyme activity, 10% (v/v) glycerol was added to subsequent samples. The reaction mixtures were prepared in 96-well plates and assayed using the Real-Time PCR Detection System according to the CFX96 Touch Protein Thermal Shift Assay Protocol manual [39].

### Cofactor determination rate and regeneration system

Oxidation of NADPH or NADH (1 mM) was monitored by in vitro reaction using a spectrophotometer (Eppendorf BioSepctrometer Kinetic) at λ = 340 nm. To measure possible peroxyflavin decoupling, the same assay was performed without substrate addition. To test the effect of flavin dinucleotide on RgF8H activity, FAD was added to the standard reaction mixture at a concentration of 0.01 mM and the reaction was carried out for 30 min. The cofactor regeneration system used recombinant N-terminally His_6×_ tagged *Bacillus megaterium* GDH [40], cloned, overexpressed, and purified in the same way as described previously in sections Bacterial strains and plasmids, Protein expression, and purification. To test the efficiency of the regeneration system, the reaction was carried out (20 µL RgF8H (1.5 mg/mL), 0.1 mM naringenin, 0.01 mM NADP^+^, 20 µL GDH (1.0 mg/mL), 0.3 mM glucose, 25 mM sodium phosphate buffer, pH 7.5) at 30 ℃, 800 rpm for 30 min.

### Substrate specificity

For substrate specificity analysis, the compounds collected in Additional file 1: Table S1 were tested at a final concentration of 0.01 mM. Each compound was added to a standard assay mixture. All samples were further analyzed as previously described for in vivo and in vitro assays.

### UPLC and LC/MS analyses

UPLC analysis was performed on an Ultimate 3000 chromatograph from Dionex (Sunnyvale, CA, USA) equipped with a DGP-3600A dual pump liquid control module, and a TCC-3200 thermostated column compartment, a WPS-3000 autosampler, and a diode array detector. The system was controlled and data acquisition was performed using Chromeleon 6.80 software (Dionex, Sunnyvale, CA, USA). Separation was performed on an Acclaim TM RSLC Polar Advantage II column (2.1 × 100 mm, 2.2 μm, Advanced Materials Technology Inc., Wilmington, DE, USA) equipped with a pre-column. A linear gradient system was used for elution using a mobile phase consisting of 0.01% formic acid in water (solvent A) and 0.01% formic acid in acetonitrile (solvent B): 0 min, 15% B; 0–4.2 min, 98% B; 4.2–6.0 15% B. The flow rate was 0.7 mL/min, the injection volume was 10 μL and the column temperature was 28 °C. Detection was carried out at 280 or 330 nm. UV–Vis spectra were measured in the range of 200–600 nm. Identification of compound peaks was based on a comparison of their retention time and UV spectrum with standard compounds.

LC–MS analysis was performed on an LC–MS 8045 SHIMADZU (SHIM-POL A.M. Borzymowski, Warsaw, Poland) equipped with a triple quadrupole, and diode array detector. The system was controlled and data were collected using LabSolutions software (Shimadzu, Kyoto, Japan). A Kinetex column C18 (3 × 100 mm, 2.6 µm 100 Å, Phenomenex, Torrance, CA, USA) equipped with a pre-column was used for the separation. The mobile phase was a mixture of water with 0.01% formic acid v/v (A) and acetonitrile (B). The program was as follows: 80% B and 20% A in 5 min. The flow rate was 0.3 mL/min, the injection volume was 2 µL, and the column temperature was 30 °C. The major operating parameters were as follows: nebulizing gas flow: 3 L/min, heating gas flow: 10 L/min, interface temperature: 300 °C, drying gas flow: 10 L/min, data acquisition range m/z 200–500 Da, ionization mode—positive and negative. Identification of compounds was based on calculated molecular mass.

### Statistical analysis

All measurements were performed in triplicate. Data are summarized as mean and standard deviation. Data were analyzed and graphed using RStudio version 1.4.1106 software.

## Results

### Induction of *R. glutinis*

**Fig. 2:**
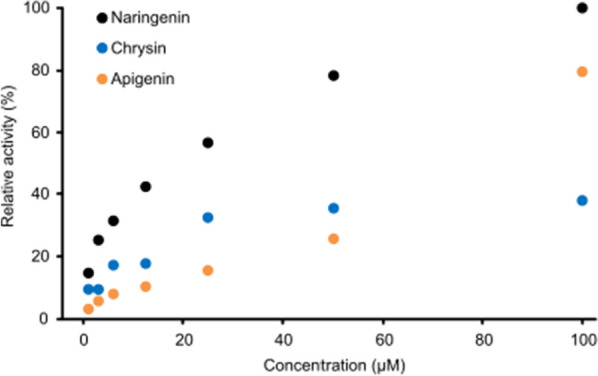
Induction of hydroxylase from *R. glutinis* by selected flavonoids, over a range of concentrations (1–100 µM)

Our work on the identification of F8H started from trails of protein purification and characterisation from yeast cultures, although reactions using crude enzymatic extracts failed, and only traces of products were detected. This prompted us to test whether the enzyme responsible for the C-8 hydroxylation is constitutive or inducible. Experiments with cycloheximide (inhibits biosynthesis of new proteins) addition clearly demonstrated that the F8H activity was induced by exposure of culture on flavonoids. In the pool of the inducers (broad panel of phenols, naphthalenes, coumarins, tetralons, flavonoids, and steroids) only flavonoids were able to induce enzymatic activity in vivo for further naringenin oxidation. The highest F8H activity was observed using naringenin, however also chrysin, and apigenin induced F8H, but less efficiently (Fig. 2). Since our effort focused on the determination of maximal activity for the planned RNA seq experiment, we have also determined the best flavonoid concentration that might provide higher transcriptome differences. The best results were obtained using flavonoid concentrations of 100 µM, although a higher concentration of inducer might work better as clearly induction is concentration-dependent.

### Identification of cDNA encoding F8H in *R. glutinis*

**Fig. 3:**
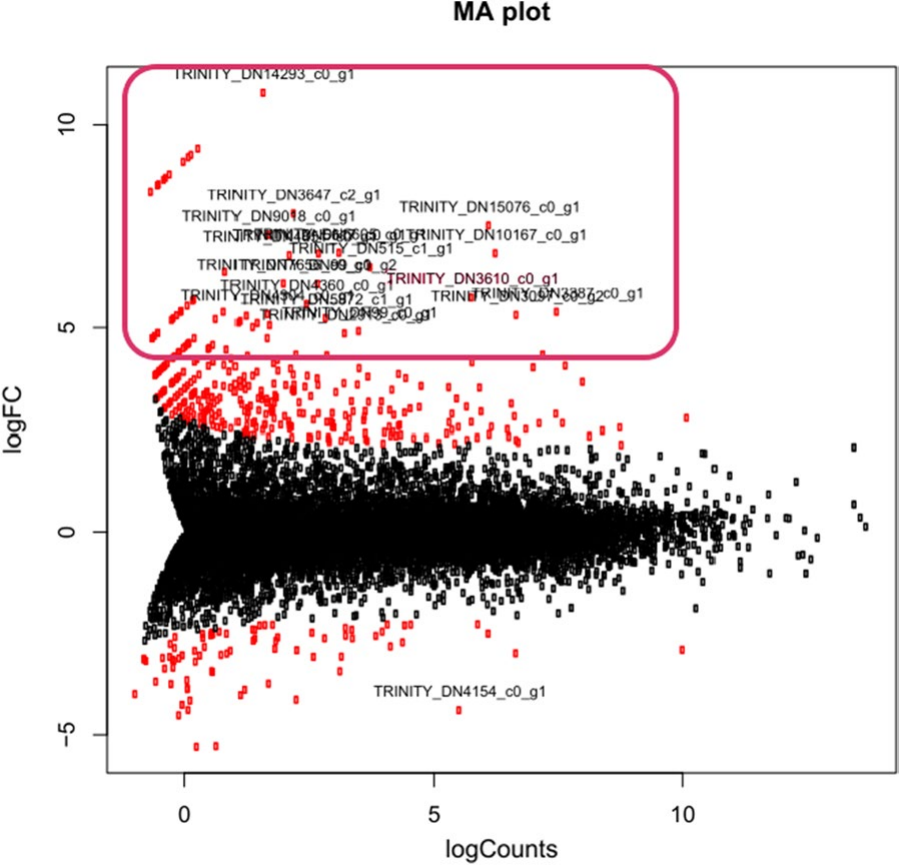
The difference in expression of individual transcripts between induced and uninduced samples, boxed transcript—hundred records with the greatest differences between the transcriptomes of induced and uninduced *R. glutinis* KCh735 cultures

By analyzing the differences between the transcriptomes of induced and uninduced *R. glutinis* KCh735 cultures using statistical analysis, the hundred records with the greatest differences were extracted (Fig. 3). The blastn program was used to check the selected records for FAD-binding sequence and homology to the hydroxylase sequence from *H. seropedicae* (fdeE) [41]. Blast analysis identified a set of homologs: Rg3610, Rg7441, Rg2726, and Rg6421. In silico analysis revealed that Rg3610 consists of two sequences Rg3610short and Rg3610long, which may be a result of alternative splicing. On this basis, 5 sequences potentially responsible for encoding C-8 hydroxylase were selected for further analysis.

### Purification of recombinant protein

**Fig. 4:**
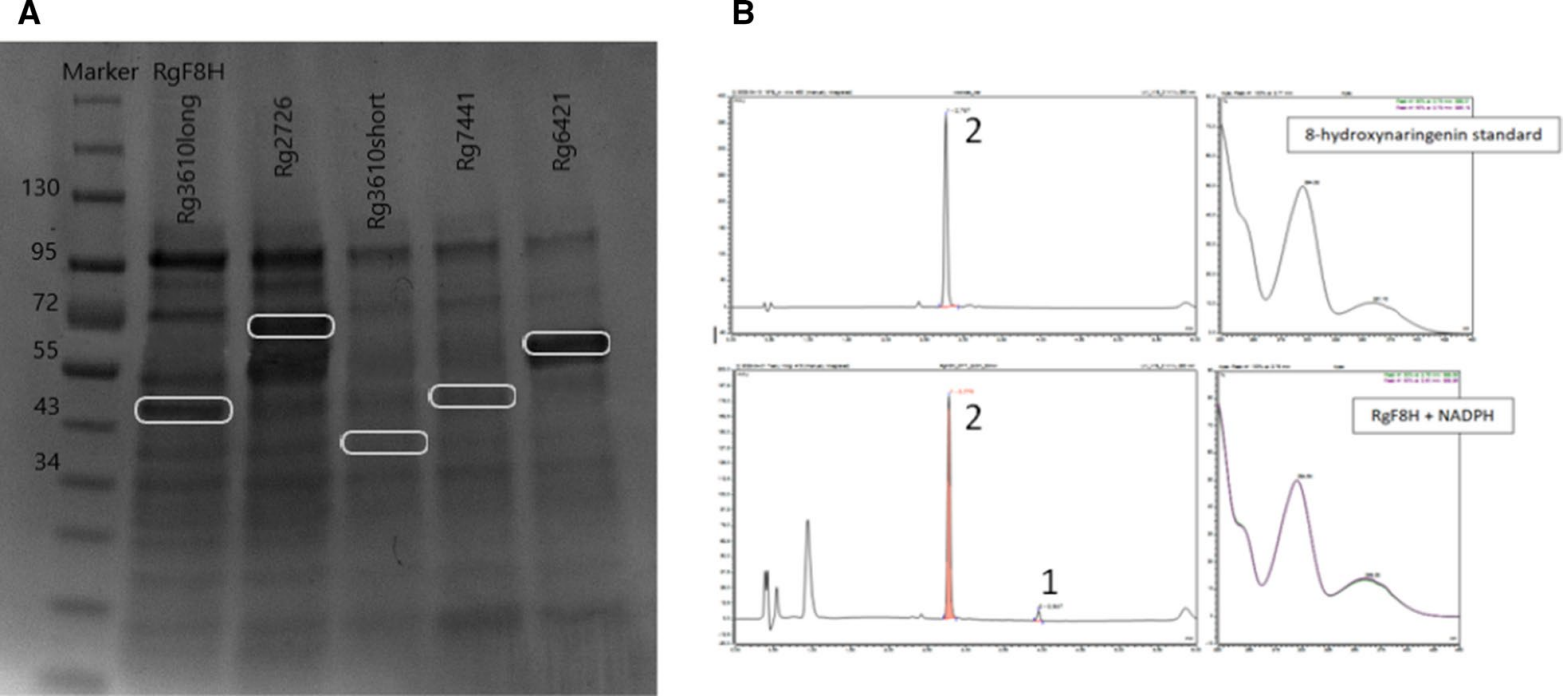
**A** Sodium dodecyl sulfate-polyacrylamide gel electrophoresis of recombinant hydroxylases from *R. glutinis* KCh735: marker (line 1), Rg3610long = RgF8H (line 2), Rg2726 (line 3), Rg3610short (line 4), Rg6421 (line 5), Rg7441 (line 6). **B** Identification of reaction products catalyzed by the recombinant protein from *R. glutinis* KCh735. The UPLC profiles were monitored by a photodiode array detector. 1: Naringenin, 2: 8-hydroxynaringenin

To verify protein overexpression, selected codon-optimized sequences were ordered and cloned into the pSEVA182 vector. Sequences were assembled to both constitutive expression using PBba_J23100 synthetic promoter (http://parts.igem.org/Promoters/Catalog/Anderson) and RhaS/PRhaBAD inducible expression systems and the recombinant proteins were overexpressed in *E. coli* BL21 (DE3) cells using an inducible expression system. IMAC Ni^2+^ affinity chromatography was used for purification. The SDS-Page analysis confirmed the ability of *E. coli* cells to express proteins from *R. glutinis* KCh735 (Fig. 4A). The highest overexpression was observed for Rg2726. Rg3610long and Rg7441 were also expressed in bacterial cells, but with lower efficiency. The remaining sequences require expression optimization.

With overexpressed proteins in hand, we performed also in vivo and in vitro assays using naringenin as the substrate that clearly demonstrate that the selected Rg3610long sequence was the enzyme we were looking for. Analysis of the reaction products by ultra-high performance liquid chromatography (UPLC) showed a peak upon 2.896 min with characteristic UV spectra, corresponding to 8-hydroxynaringenin (Fig. 4B). The positive control 8-hydroxynaringenin was also eluted at 2.896 min. The results indicated that 8-hydroxynaringenin is the product of RgF8H in DH5α cells and that RgF8H is Rg3610long transcript. Other sequences did not show any activity for naringenin. However, given their high level of transcription in naringenin-induced cultures and the possibility of protein expression in bacterial cells, they require further study, which is beyond the scope of this paper.

### Phylogenetic analysis

**Fig. 5:**
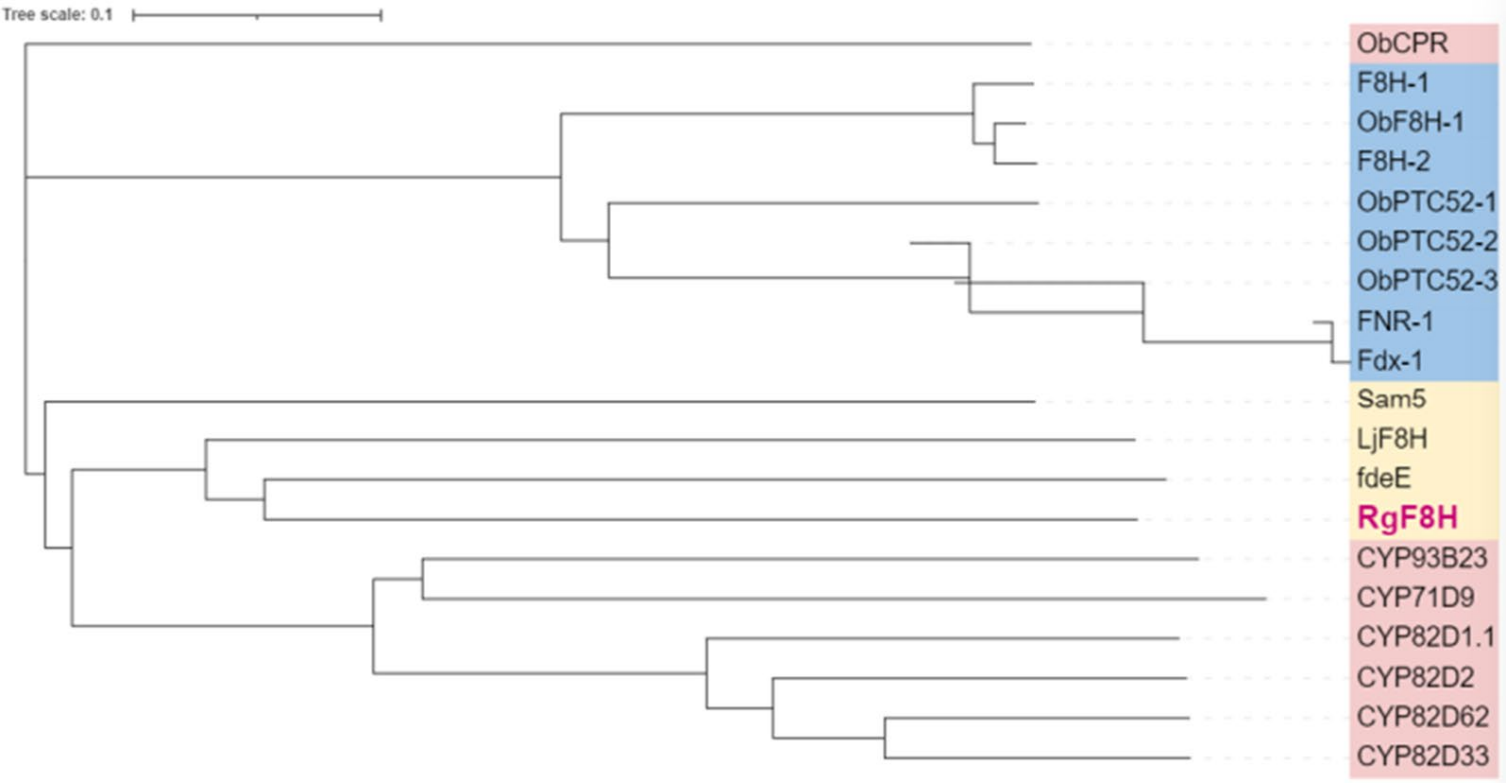
Phylogenetic analysis based on C-6, C-8, and Riske-type hydroxylase sequences. Protein sequences downloaded from the National Center for Biotechnology Information (NCBI) were aligned using Clustal Omega software (https://www.ebi.ac.uk/Tools/msa/clustalo). The phylogenetic tree was constructed using iTOL software (https://itol.embl.de/). Bootstrap values are given next to each branch. The line length indicates the evolutionary distance calculated by the Poisson correction method, and the branch labels are the protein names. Labels used: blue colour—Riske enzymes, pink colour—C-6 hydroxylases, yellow colour—C-8 hydroxylases. The nucleotide sequences of the proteins used in this study are available in the Additional file 1: Table S3

RgF8H shares low sequence identity with fdeE (29.26%) and LjF8H (*Lotus japonicus*) (24.14%). However, a phylogenetic tree constructed among known F8H [21, 24, 34, 42], and F6H [21, 23, 43] (Fig. 5) grouped RgF8H (Rg3610long) under C-8 monooxygenases, suggesting that RgF8H is a flavin-containing monooxygenase (FMO), requiring NADPH and/or FAD for its activity. Since fdeE from *H. seropedicae* is known to require FAD for its activity, it was predicted that RgF8H from *R. glutinis* KCh725 also might exhibit FAD-dependent activity. Sequence searches for the FAD-binding motif (GxGxxG), F motif (FxGxxxHxxxy), and GD motif (GDAxHxxxPxxxxG) were performed using SnapGene software version 5.1.7 (standard conditions). The analysis confirmed the presence of an N-terminal GxGxxG sequence and a C-terminal GDAxHxxxPxxxxG sequence in Rg3610long. Both these sequences were also identified for fdeE (Additional file 1: Figure S1). The identification of a highly conserved FAD and GD motif for FAD-dependent oxidases suggests that RgF8H is also a FAD-dependent oxygenase. However, the Rg3610long (RgF8H) sequence is devoid of the F motif. Interestingly, the fdeE and LjF8H [24] sequences also do not contain an F motif, suggesting that all these enzymes may belong to a new FMO group.

### Enzyme activity and cofactor effects of recombinant RgF8H

**Fig. 6:**
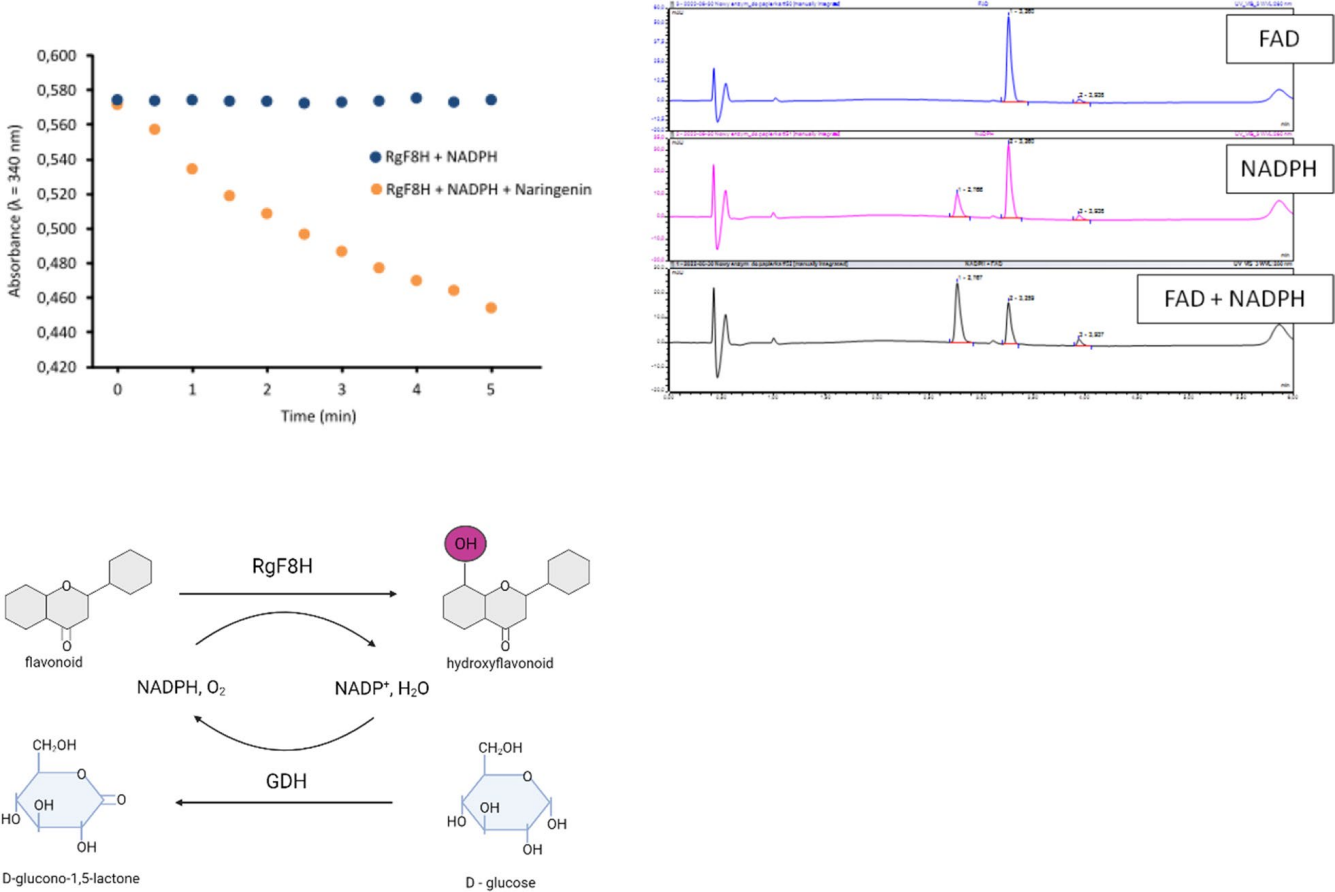
**A** Oxidation of NADPH by RgF8H in presence of naringenin and without substrate addition. **B** Effect of FAD on RgF8H activity. **C** NADPH cofactor regeneration system essential for RgF8H activity

Evaluated flavonoids hydroxylases are flavin-containing enzymes, therefore a key parameter to consider for future applications is to evaluate the decupling rate of the peroxyflavin intermediate formed upon reaction with molecular oxygen. The decupling rate can be determined simply by measurement of NADPH oxidation, after the initial saturation of the reaction buffer with oxygen. In the case of purified RgF8H, the NADPH concentration did not change for 30 min of incubation, indicating an almost complete absence of decoupling, thus no production of hydrogen peroxide. Here is a vital point, as the hydrogen peroxide produced in the host could damage the cell, and oxidation of both substrate and product would probably occur. A comparison of cofactor utilization showed that both NADPH and NADH concentrations decrease when incubated with naringenin. NADH was utilized virtually immediately but UPLC–DAD analysis showed that this was not associated with product formation. The high transformation of NADH is probably due to the background activity present in the purified RgF8H fraction (Fig. 4A). Application of NADPH and related concentration drop was much slower, however, allowed the product to be observed via UPLC–DAD. For that reason, a comparative cofactor oxidation assay was performed for purified RgF8H with and without substrate addition. Figure 6A clearly shows that NADPH is oxidized, and flavin is only reduced in the presence of naringenin. This assay shows that RgF8H enzyme is NADPH-dependent monooxygenase. In addition, it was verified whether the presence of FAD affected enzyme activity. The results indicated that the addition of FAD is not necessary, but significantly improves the efficiency of RgF8H activity. After 30 min of reaction, a 24.4% conversion of naringenin was observed for the sample with NADPH, while the use of both NADPH and FAD allowed more than twofold increase in substrate conversion (58.2%) (Fig. 6B).

Due to the fact that the cost of NADPH is extremely high, it was essential to develop a regeneration system. The previous work confirmed that the glucose dehydrogenase (GDH) gene from *Bacillus megaterium* is overexpressed in *E. coli* cells [40]. It was concluded that the resulting recombinant GDH could act as an NADPH regeneration system, similar to commercially available GDH [44] (Fig. 6C). For this purpose, Bm_GDH was cloned, overexpressed, and purified in the same way as RgF8H. The use of a regeneration system resulted in high substrate conversion, with no substrate detected after 30 min of reaction. A reaction using NADPH as an electron donor carried out under the same conditions led to the conversion of 81% of the substrate. The presence of Bm_GDH ensuring continuous regeneration of the cofactor allows the reaction to proceed until the substrate is depleted. This indicates that the absence of a cofactor in the reaction mixture stops the reaction, so it is crucial for the efficient formation of hydroxy-derived flavonoids.

### Biochemical assays

**Fig. 7:**
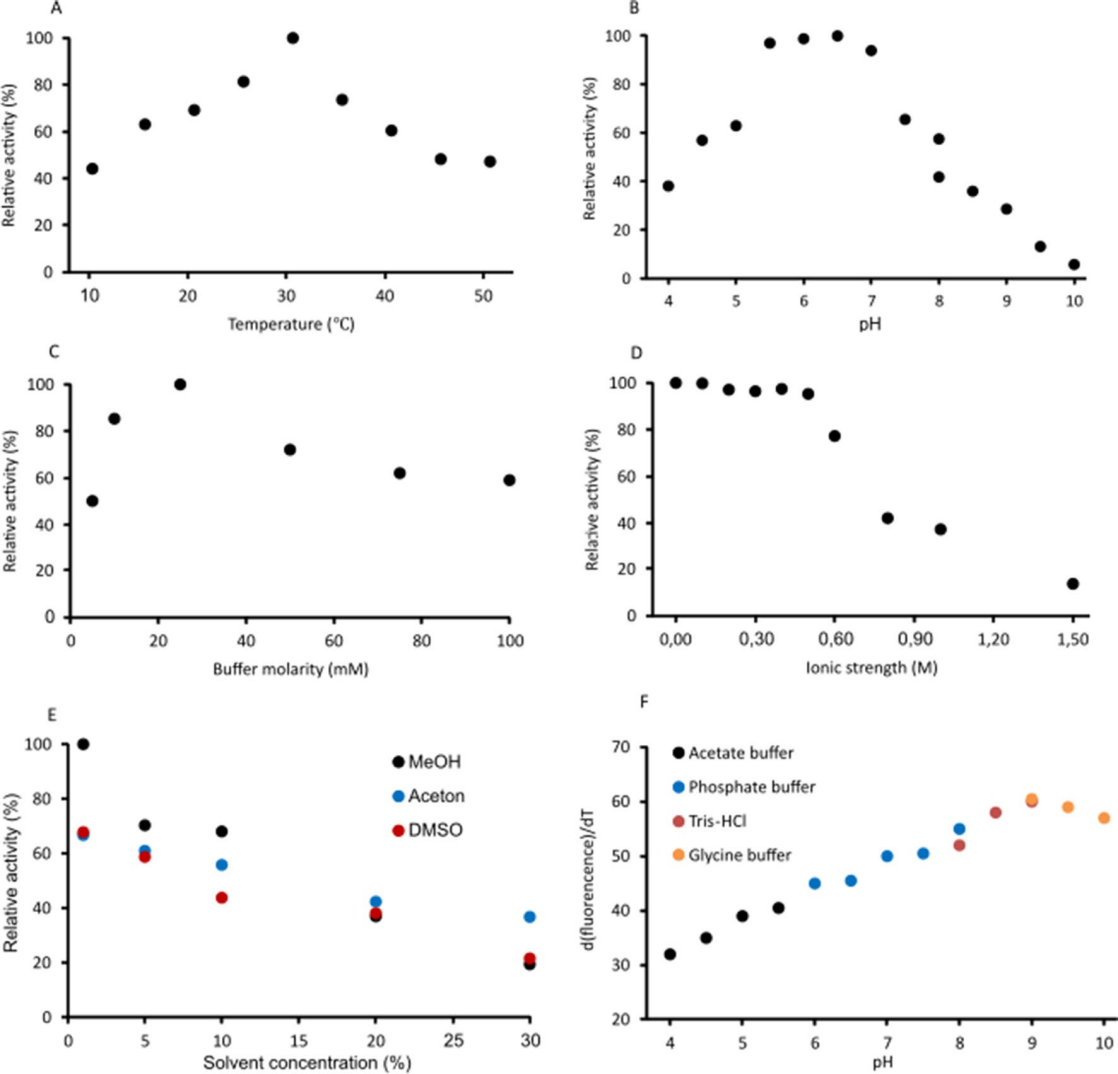
Analysis of the biochemical properties of RgF8H in vitro. Data in all panels represent averages over three replicates. The standard deviations obtained were < 3% of relative activity and were not included in the point markers. Effect of **A** temperature, **B** pH, **C** buffer molarity, **D** ionic strength, and **E** solvent concentration on RgF8H activity. **F** Stability of RgF8H analyzed via ThermoFAD under different conditions

The basic characterization of the reaction conditions for the enzyme was assessed by measuring the NADPH concentration. The purified recombinant RgF8H was evaluated for biochemical aspects such as temperature, pH, buffer, ionic strength, and stability. The enzyme showed a wide temperature optimum (Fig. 7A). The highest relative activity (above 90%) was found at 25–30 °C. The activity was not detected at temperatures above 54 °C and below 20 °C. We also evaluated the effect of reaction pH on RgF8H activity (Fig. 7B). The enzyme showed a narrow range in which the enzyme activity was within 90% (around pH 5.0–5.5), although the enzyme denatures rapidly under these conditions. The ThermoFAD assay showed that RgF8H was denatured in the buffer of pH 5.5 and lower in the temperature range of 32–40 °C (Fig. 7F). The use of a more alkaline environment significantly increased the stability of the enzyme. The use of a pH 6–6.5 buffer resulted in denaturation of the enzyme at 45 ℃, while in a pH 7–7.5 the enzyme cleaved at 50 ℃. The enzyme was most stable in the pH 8–10 range (denaturation at 52–60 ℃) Interestingly, it was observed that the type of buffer also influenced the stability of the protein (pH = 8, phosphate buffer—55 ℃, Tris–HCl buffer—52 ℃). Consequently, a phosphate buffer of pH 7 was used in further studies, in which it was both stable (50 ℃) and very active (94% substrate conversion). However, the addition of 10% glycerol to the mixture had a noticeable effect on the stability of the enzyme. After evaluating the above parameters, the focus was on the choice of reaction buffer to maximize RgF8H activity in vitro. The molarity of the buffers used did not have an as noticeable effect on enzyme activity as the previously studied parameters (Fig. 7C), although the best results were observed in the 10–25 mM range. Regarding ionic strength, a decrease in the relative activity below 90% was observed at salt concentrations of 0.8 M and above (Fig. 7D). The addition of a small amount of DMSO (0–10%) as a co-solvent had a beneficial effect on the (Fig. 7E).

### Substrate specificity and structural identification of hydroxylation products

The in vivo reaction was carried out with 30 different compounds (flavonols, flavones, flavanones, isoflavones, and chalcones) under identical conditions as reported in the methods section. The reaction mixture was analyzed by ultra-high-performance liquid chromatography with a diode array (UPLC–DAD) for preliminary analysis of hydroxylation products. Of the 30 substrates tested, nine flavonoids (naringenin, eriodictyol, pinocembrin, hesperetin, chrysin, apigenin, luteolin, diosmetin, 7,4ʹ-dihydroxyflavone) were hydroxylated with RgF8H. Comparative percentages of conversion of each substrate to products using monooxygenase from *R. glutinis* KCh735 are shown in Table 1. Each reaction product was characterized by UV absorbance maxima and authentic standards obtained in previous studies [11], and by high-resolution quadruple electrospray ionization (HR-QTOF ESI/MS), as shown in Table 1 and Additional file 1. Among the flavanones, the conversion of naringenin to the hydroxylated derivative (retention time for peak 1 (t_R_) ~ 2.778 min, Fig. 3B; calculated mass for the molecular formula C_15_H_12_O_6_ for [M+H]^+^ *m/z*^+^ ~ 289. 25, [M−H]^−^ *m/z*^−^ ~ 287. 25, for which the observed masses are ~ 289 and 287, respectively, λ_max_: 293 nm) (Additional file 1: Figure S2) by RgF8H was 63.84% compared to 100% conversion achieved in vitro by purified protein in combination with a GDH regeneration system (Fig. 6C). Similarly, the conversion of eriodictyol and pinocembrin to hydroxylated products using recombinant RgF8H proceeded in high yields of 65% and 62%, respectively (8-hydroxyeriodictyol: calculated mass for molecular formula C_15_H_12_O_7_ for [M+H]^+^ *m/z*^+^ ~ 305.25 and [M−H]^−^ *m/z*^−^ ~ 302.25, for which the mass was observed [M+H]^+^ *m/z*^+^ ~ 305 and [M−H]^−^ *m/z*^−^ ~ 303, λ_max_: 292 nm, 8-hydroxypinocembrin: calculated mass for the molecular formula C_15_H_12_O_5_ for [M+H]^+^ *m/z*^+^ ~ 273.25 and [M−H]^−^ *m/z*^−^ ~ 271.25, for which mass [M+H]^+^ *m/z*^+^ ~ 273 and [M−H]^−^ *m/z*^−^ ~ 271, λ_max_: 295 nm) was observed (Additional file 1: Figures S3 and S4). RgF8H allowed to obtain the product of hesperetin oxidation (calculated mass for the molecular formula C_16_H_14_O_7_ for [M+H]^+^ *m/z*^+^ ~ 319.27 and [M−H]^−^ *m/z*^−^ ~ 317.27, for which [M+H]^+^ *m/z*^+^ ~ 319 and [M−H]^−^ *m/z*^−^ ~ 317, λ_max_: 292 nm) with a conversion of 30.64% (Additional file 1: Figure S5). Similar hydroxylation reaction was also observed for flavones: luteolin (calculated mass for the molecular formula C_15_H_10_O_7_ for [M+H]^+^ *m/z*^+^ ~ 303.24 and [M+H]^−^ *m/z*^−^ ~ 301.24, for which mass was observed [M+H]^+^ *m/z*^+^ ~ 303 and [M+H]^−^ *m/z*^−^ 301, λ_max_: 278, 338 nm) and apigenin (calculated mass for molecular formula C_15_H_10_O_6_ for [M+H]^+^ *m/z*^+^ ~ 287.24 [M+H]^−^ *m/z*^−^ ~ 285.24, for which masses of [M+H]^+^ *m/z*^+^ ~ 289 and [M+H]^−^ *m/z*^−^ 287 were observed, λ_max_: 280, 303 nm) with conversions of 73.09% and 20.02%, respectively (Additional file 1: Figures S6 and S7). In contrast, for 8-hydroxychrysin (calculated mass for the molecular formula C_15_H_10_O_5_ for [M+H]^+^ *m/z*^+^ ~ 271.24 and [M+H]^−^ *m/z*^−^ ~ 269.24, for which the observed mass of [M+H]^+^ *m/z*^+^ ~ 271 and [M+H]^−^ *m/z*^−^ 269, λ_max_: 280), 8-hydroxydiosmetin (calculated mass for the molecular formula C_16_H_12_O_7_ for [M+H]^+^ *m/z*^+^ ~ 317.26 and [M+H]^−^ *m/z*^−^ ~ 315.26, for which the mass of [M+H]^+^ *m/z*^+^ ~ 317 and [M+H]^−^ *m/z*^−^ 315, λ_max_: 279 and 336 nm) and 7,8,4ʹ-trihydroxyflavone (calculated mass for the molecular formula C_15_H_10_O_5_ for [M+H]^+^ *m/z*^+^ ~ 271.23 and [M+H]^−^ *m/z*^−^ ~ 269.23, for which the mass of [M+H]^+^ *m/z*^+^ ~ 271 and [M+H]^−^ *m/z*^−^ 269, λ_max_: 288 nm) the observed conversion was much lower, 8.92, 7.38 and 1.8%, respectively (Additional file 1: Figures S8–S10). NMR analysis of the presented C-8 hydroxy-derivatives was described in an earlier publications [26, 27].

**Table 1:** Product of conversion, UPLC–DAD, HR-QTOF ESI/MS analysis, and UV maxima of substrates in vivo reaction using RgF8H

Substrates					Products					
Name	UPLC (t_R_) [min]	Mass		UV maxima [nm]	Name	Conversion (%)	UPLC (t_R_) [min]	Mass		UV maxima [nm]
		[M+H]^+^ *m/z*^+^	[M−H]^−^ *m/z*^−^					[M+H]^+^ *m/z*^+^	[M−H]^−^ *m/z*^−^	
Naringenin	3.271	273	271	288.90	8-hydroxynaringenin	63.84	2.768	289	287	293.52
Eriodictyol	2.993	289	287	287.98	8-hydroxyeriodictyol	64.94	2.454	305	303	291.78
Pinocembrin	3.623	257	255	289.13	8-hydroxypinocembrin	61.61	3.121	273	271	294.62
Hesperetin	3.212	303	301	287.43	8-hydroxyhesperetin	30.64	2.761	319	317	292.35
Luteolin	3.245	287	285	252.66, 346.00	8-hydroxyluteolin	73.09	2.781	303	301	278.55, 338.04
Apigenin	3.550	271	269	267.38, 334.78	8-hydroxyapigenin	20.02	3.039	289	287	279.79, 303.08
Chrysin	3.792	255	253	267.31	8-hydroxychrysin	8.92	3.281	271	269	280.01
Diosmetin	3.409	301	299	250.98, 343.60	8-hydroxydiosmetin	7.38	2.955	317	315	279.77, 336.07

The percentage of conversion was calculated based on the decrease of substrates, due to the instability of the obtained hydroxyflavonoid derivatives

The RgF8H was active towards flavanones, and flavones. Noteworthy is the position of hydroxyl groups in the A ring of the substrate. Analysis of substrate specificity demonstrates the requirement of the C-7 hydroxyl group for the enzymatic activity, justifying the *ortho*-hydroxylation reaction mechanism.

### GenBank accession number

The raw sequence reads from the analysis of the *Rhodotorula glutinis* KCh735 transcriptome used in this study are deposited as BioProject accession number PRJNA859513. The nucleotide sequences of the proteins used in this study (Additional file 1: Table S3) were derived from the sequencing of the transcriptome deposited as BioProject accession number PRJNA859513.

## Discussion

*Rhodotorula glutinis* KCh735 and *Rhodotorula marina* AM77 [25] are the only yeast described in the literature capable of regioselective *ortho*-hydroxylation of the A-ring of flavonoids [27]. Presented transformations of flavones and flavanones allow to produce interesting natural molecules in simple, cheap, and, most importantly, specific ways. The coding sequence of F8H responsible for the hydroxylation of flavones and flavanones in *R. glutinis* KCh735 has not been known so far. Hydroxylation of secondary metabolites (flavonoids) is one of the major modifications that have profound effects on physical and biological changes in molecules [45]. In this study, the sequence encoding a flavin-dependent monooxygenase from *R. glutinis* KCh735 was identified and characterized biochemically and substrate-wise in relation to flavonoid aglycones. Oxidative hydroxylation of naringenin was shown by only one of the sequences tested, which accepted various flavonoids as substrates for hydroxylation, including flavones (apigenin, chrysin, luteolin, diosmetin, 7,4ʹ-dihydroxyflavone), and flavanones (naringenin, pinocembrin, hesperetin, eriodictyol) (Table 1). These results indicate that enzymatic synthesis using *E. coli* cells carrying recombinant proteins has provided prospects for the synthesis of valuable hydroxylated flavonoids.

RgF8H shares most of the biochemical parameters with other FAD-dependent enzymes described in the literature. It has a FAD-binding motif, suggesting that it is a FAD-dependent monooxygenase (Additional file 1: Figure S1). However, an in vitro study showed that RgF8H absolutely requires NADPH for its activity, although we do not find an NADP^+^-binding motif in the sequence. Similarly, LjF8H also showed an absolute requirement for NADPH [24]. It is debatable whether these enzymes do not require FAD for their activity or whether they bind FAD strictly during expression and do not release it during sample preparation. The addition of flavin mononucleotide or FAD did not stimulate the initial rate of the enzymatic reaction of taxifolin 8-monooxygenase, suggesting tight binding of FAD to the enzyme [46]. This result supports the researcher’s hypothesis that FAD may also remain tightly bound to LjF8H. The opposite results were obtained for RgF8H, which was also not active in the presence of FAD alone, but the addition of FAD to the reaction mixture containing the NADPH coenzyme increased doubles the reaction efficiency.

The described *H. seropedicae* genes relevant to the flavonoid degradation pathway are organised in an operon consisting of 10 orf. Naringenin and chrysin strongly induced fdeA::lacZ fusion expression, suggesting that this group of genes is involved in the degradation of naringenin and probably related flavonoids. The high homology between RgF8H and fdeE suggests that a similar gene arrangement may also exist in the yeast genome. The analysis of a potentially occurring operon in *R. glutinis* cells involved in the degradation of flavonoid compounds seems to be a promising direction for further research. The fdeE from *H. seropedicae* [41] shows activity towards naringenin (flavanone), there are no reports of attempts to use this enzyme against other flavonoids. In contrast, LjF8H [24] shows broad activity against flavanones, flavones, flavanonols, and flavonols. F8H from *Chrysanthemum segetum* has been characterized as a cytochrome P450 monooxygenase and hydroxylates luteolin and quercetin [47]. ObF8H-1 shows high specificity towards flavones: salvigenin and cirsimaritin [42], and SbCYP82D2 is active towards chrysin (flavone) but not towards other flavones or flavanones [47], while Sam5 shows activity towards flavones, flavanones, flavonols, and isoflavones [48]. In the case of RgF8H the only accepted substrates are flavanones and flavones. Furthermore, the use of a cofactor regeneration system involving the GDH enzyme from *B. megaterium* increased the reaction efficiency and allowed 100% conversion of naringenin to be achieved within 30 min.

The unique catalytic properties of oxygenases (e.g. regio-specific hydroxylation of inactive carbons) are of undisputed biosynthetic value. However, there are many obstacles to their large-scale practical application, which include high enzyme instability and low efficiency of expression of components building the structure of oxygenases in *E. coli* [49, 50]. Only the bacterial monooxygenase Sam5 is well expressed in *E. coli*, but it performs the biotransformation of naringenin to three products [48]. Flavonoid 8- and 6-hydroxylases (CYP82D2 and CYP82D1, respectively) from *Scutellaria baicalensis*, active against chrysin, were efficiently expressed in WAT11 yeast cells [21], with no data for *E. coli* expression. CYP71D9—a flavonoid 6-hydroxylase from *Glycine max*, characterized as a P-450 monooxygenase, was also efficiently expressed in yeast *Saccharomyces cerevisiae* cells [23]. RgF8H is the only monooxygenase so far described with such conservative regioselectivity towards flavonoids.

## Conclusions

The present study demonstrates the utility of simple differential expression analysis in order to identify the sequence of RgF8H gene. The enzyme accepts naringenin, eriodictyol, pinocembrin, hesperetin, luteolin, apigenin, chrysin, diosmetin, and 7,4ʹ-dihydroxyflavone as substrates resulting in corresponding C8-OH derivatives. The C8-hydroxylase activity tightly linked to the presence of a C-7 hydroxyl group, which explains the *ortho*-hydroxylation mechanism. For its activity, RgF8H requires the presence of NADPH coenzyme, which is an expected feature of FAD-dependent monooxygenases. High activity of the enzyme in *E. coli* host provides strong background for further application of the in synthetic biology driven production of rare and highly antioxidant flavonoids.

## Supplementary Information

**Additional file 1: Table S1. **Structures of compounds related to this work. **Table S2.** List of compounds used as potential C-8 hydroxylase inducers. **Table S3.** Accession number and origin of sequences used in the phylogenetic analysis. **Table S4.** Sequences of flavonoid C-8 hydroxylase gene candidates, primer sequences and transcription units. **Figure S1.** Nucleotide sequences encoding the amino acids fdeE—line 1, and Rg3610long (RgF8H)—line 2. Rg6421, Rg2726. Highly conserved FAD and GD motifs were highlighted in boxes using red and blue underscores, respectively. **Figure S2.** LC–MS analysis of naringenin hydroxylation by RgF8H. (A) Mass spectrum of naringenin, (B) 8-hydroxynaringenin. **Figure S3.** LC–MS analysis of eriodictyol hydroxylation by RgF8H. (A) Mass spectrum of eriodictyol, (B) 8-hydroxyeriodictyol. **Figure S4.** LC–MS analysis of pinocembrin hydroxylation by RgF8H. (A) Mass spectrum of pinocembrin, (B) 8-hydroxypinocembrin. **Figure S5.** LC–MS analysis of hesperetin hydroxylation by RgF8H. (A) Mass spectrum of hesperetin, (B) 8-hydroxyhesperetin. **Figure S6.** LC–MS analysis of luteolin hydroxylation by RgF8H. (A) Mass spectrum of luteolin (B) 8-hydroxyluteolin. **Figure S7.** LC–MS analysis of apigenin hydroxylation by RgF8H. (A) Mass spectrum of apigenin, (B) 8-hydroxyapigenin. **Figure S8.** LC–MS analysis of chrysin hydroxylation by RgF8H. (A) Mass spectrum of chrysin, (B) 8-hydroxychrysin. **Figure S9.** LC–MS analysis of diosmetin hydroxylation by RgF8H. (A) Mass spectrum of diosmetin, (B) 8-hydroxydiosmetin. **Figure S10.** LC–MS analysis of 7,4ʹ-dihydroxyflavone hydroxylation by RgF8H. (A) Mass spectrum of 7,4ʹ-dihydroxyflavone, (B) 7,8,4ʹ-trihydroxyflavone.

## Data Availability

Not applicable.

## Abbreviations

F6H: Flavonoids 6-hydroxylase
F8H: Flavonoids 8-hydroxylase
RgF8H: Flavonoid 8-hydroxylase from *Rhodotorula glutinis* KCh735
fdeE: Hydroxylase from *Herbaspirillum seropedicae*
NADPH: Nicotinamide adenine dinucleotide phosphate
NADH: Nicotinamide adenine dinucleotide
FAD: Flavin adenine dinucleotide
SBS: Sequencing by synthesis
LB: Lysogeny broth
UPLC–DAD: Ultra high-performance liquid chromatography with diode-array detector
DMSO: Dimethyl sulfoxide
LC–MS: Liquid chromatography with mass spectrometry
MeOH: Methanol
