# Supplementary material for: Novel flavonoid C-8 hydroxylase from Rhodotorula glutinis: identification, characterization and substrate scope
Source: Microb Cell Fact. 2022 Aug 29;21:175. doi: 10.1186/s12934-022-01899-x (PMC9422121; doi:10.1186/s12934-022-01899-x)
Supplement: Supplementary file 1 — Additional file 1: Table S1. Structures of compounds related to this work. Table S2. List of compounds used as potential C-8 hydroxylase inducers. Table S3. Accession number and origin of sequences used in the phylogenetic analysis. Table S4. Sequences of flavonoid C-8 hydroxylase gene candidates, primer sequences and transcription units. Figure S1. Nucleotide sequences encoding the amino acids fdeE—line 1, and Rg3610long (RgF8H)—line 2. Rg6421, Rg2726. Highly conserved FAD and GD motifs were highlighted in boxes using red and blue underscores, respectively. Figure S2. LC–MS analysis of naringenin hydroxylation by RgF8H. (A) Mass spectrum of naringenin, (B) 8-hydroxynaringenin. Figure S3. LC–MS analysis of eriodictyol hydroxylation by RgF8H. (A) Mass spectrum of eriodictyol, (B) 8-hydroxyeriodictyol. Figure S4. LC–MS analysis of pinocembrin hydroxylation by RgF8H. (A) Mass spectrum of pinocembrin, (B) 8-hydroxypinocembrin. Figure S5. LC–MS analysis of hesperetin hydroxylation by RgF8H. (A) Mass spectrum of hesperetin, (B) 8-hydroxyhesperetin. Figure S6. LC–MS analysis of luteolin hydroxylation by RgF8H. (A) Mass spectrum of luteolin (B) 8-hydroxyluteolin. Figure S7. LC–MS analysis of apigenin hydroxylation by RgF8H. (A) Mass spectrum of apigenin, (B) 8-hydroxyapigenin. Figure S8. LC–MS analysis of chrysin hydroxylation by RgF8H. (A) Mass spectrum of chrysin, (B) 8-hydroxychrysin. Figure S9. LC–MS analysis of diosmetin hydroxylation by RgF8H. (A) Mass spectrum of diosmetin, (B) 8-hydroxydiosmetin. Figure S10. LC–MS analysis of 7,4ʹ-dihydroxyflavone hydroxylation by RgF8H. (A) Mass spectrum of 7,4ʹ-dihydroxyflavone, (B) 7,8,4ʹ-trihydroxyflavone. [file 12934_2022_1899_MOESM1_ESM.docx]

Additional file

Novel flavonoid *C-8* hydroxylase from *Rhodotorula glutinis* – identification, characterization, and substrate scope

Kinga Dulak^1,^, Sandra Sordon^1^, Agata Matera^1^, Bartosz Kozak^2^, Ewa Huszcza^1^, Jarosław Popłoński^1^

^1^ Department of Food Chemistry and Biocatalysis, Wroclaw University of Environmental and Life Sciences, Wroclaw, Poland (kinga.dulak@upwr.edu.pl), (agata.matera@upwr.edu.pl (A.M.); [sandra.sordon@upwr.edu.pl](mailto:sandra.sordon@upwr.edu.pl) (S.S.); [ewa.huszcza@upwr.edu.pl](mailto:ewa.huszcza@upwr.edu.pl) (E.H.)

^2^ Department of Genetics, Plant Breeding and Seed Production, Wroclaw University of Environmental and Life Science, Wroclaw, Poland [bartosz.kozak@upwr.edu.pl](mailto:bartosz.kozak@upwr.edu.pl) (B.K.)

Correspondence: jaroslaw.poplonski@upwr.edu.pl tel.: +48 71 320 50 19

List of content

[Table S1. Structures of Compounds related to this work 2](#_Toc107332303)

[Table S2. List of Compounds used as potential C-8 hydroxylase inducers 5](#_Toc107332304)

[Table S3. Accession number and origin of sequences used in the phylogenetic analysis. 6](#_Toc107332305)

[Table S4. Sequences of flavonoid C-8 hydroxylase gene candidates, primer sequences and transcription units. 6](#_Toc107332306)

[Figure S1. Nucleotide sequences encoding the amino acids fdeE - line 1, and Rg3610long (RgF8H) - line 2. Rg6421, Rg2726. Highly conserved FAD and GD motifs were highlighted in boxes using red and blue underscores, respectively. 7](#_Toc107332307)

[Figure S1. LC-MS analysis of naringenin hydroxylation by RgF8H. (A) Mass spectrum of naringenin, (B) 8-hydroxynaringenin. 7](#_Toc107332308)

[Figure S2. LC-MS analysis of eriodictyol hydroxylation by RgF8H. (A) Mass spectrum of eriodictyol, (B) 8-hydroxyeriodictyol. 8](#_Toc107332309)

[Figure S3. LC-MS analysis of pinocembrin hydroxylation by RgF8H. (A) Mass spectrum of pinocembrin, (B) 8-hydroxypinocembrin. 8](#_Toc107332310)

[Figure S4. LC-MS analysis of hesperetin hydroxylation by RgF8H. (A) Mass spectrum of hesperetin, (B) 8-hydroxyhesperetin. 8](#_Toc107332311)

[Figure S5. LC-MS analysis of luteolin hydroxylation by RgF8H. (A) Mass spectrum of luteolin (B) 8-hydroxyluteolin. 9](#_Toc107332312)

[Figure S6. LC-MS analysis of apigenin hydroxylation by RgF8H. (A) Mass spectrum of apigenin, (B) 8-hydroxyapigenin. 9](#_Toc107332313)

[Figure S7. LC-MS analysis of chrysin hydroxylation by RgF8H. (A) Mass spectrum of chrysin, (B) 8-hydroxychrysin. 9](#_Toc107332314)

[Figure S8. LC-MS analysis of 7,4’-dihydroxyflavone hydroxylation by RgF8H. (A) Mass spectrum of 7,4’-dihydroxyflavone, (B) 7,8,4’-trihydroxyflavone. 10](#_Toc107332315)

Table S1. Structures of Compounds related to this work.

| Compounds related to this work | Structure |
| --- | --- |
| Naringenin |  |
| Hesperetin |  |
| 2’-hydroxyflavanone |  |
| 3’-hydroxyflavanone |  |
| 4’-hydroxyflavanone |  |
| 6-hydroxyflavanone |  |
| 7-hydroxyflavanone |  |
| Isoxanthohumol |  |
| Pinocembrin |  |
| Eriodictyol |  |
| Chrysin |  |
| Baicalein |  |
| Diosmetin |  |
| Apigenin |  |
| Luteolin |  |
| 3-hydroxyflavone |  |
| 4’,7-dihydroxyflavone |  |
| Fisetin |  |
| Quercetin |  |
| Myricetin |  |
| Morin |  |
| Dihydromyricetin |  |
| Epicatechin |  |
| Genistein |  |
| Biochanin A |  |
| Daidzein |  |
| Xanthohumol |  |
| α,β-dihydroxanthohumol |  |
| Phloretin |  |
| Resveratrol |  |

# Table S2. List of Compounds used as potential C-8 hydroxylase inducers

| Nr | Potential inducer |
| --- | --- |
| 1 | Chrysin |
| 2 | Biochanin A |
| 3 | Genistein |
| 4 | Daidzein |
| 5 | Quercetin |
| 6 | 5-hydroxyflavanone |
| 7 | 6-hydroxyflavanone |
| 8 | 7-hydroxyflavanone |
| 9 | 2’-hydroxyflavanone |
| 10 | 3’-hydroxyflavanone |
| 11 | 4’-hydroxyflavanone |
| 12 | Naringenin |

# Table S3. Accession number and origin of sequences used in the phylogenetic analysis.

| Name | Origin | Acc. Number |
| --- | --- | --- |
| fdeE | *Herbaspirillum seropedicae SmR1* | ADJ62524 |
| LjF8H | *Lotus japonicus* | BBN79890 |
| CYP82D2 | *Scutellaria baicalensis* | ASW21052 |
| F8H-1 | *Ocimum basilicum* | AII16849 |
| F8H-2 | *Ocimum basilicum* | AII16848 |
| Fdx-1 | *Ocimum basilicum* | AII16854 |
| FNR-1 | *Ocimum basilicum* | AII16855 |
| ObPTC52-1 | *Ocimum basilicum* | AII16851 |
| ObF8H-1 | *Ocimum basilicum* | AII16850 |
| ObPTC52-3 | *Ocimum basilicum* | [AII16852](https://www.ncbi.nlm.nih.gov/protein/669204772) |
| ObPTC52-2 | *Ocimum basilicum* | AII16853 |
| Sam5 | *Saccharothrix espanaensis* | ABC88666 |
| AsB510 | *Azospirillum sp.* | WP_012977496 |
| CYP82D1.1 | [*Scutellaria baicalensis*](https://www.ncbi.nlm.nih.gov/Taxonomy/Browser/wwwtax.cgi?id=65409) | ASW21050 |
| CYP71D9 | *Glycine max* | NP_001304582 |
| ObCPR | *Ocimum basilicum* | [AGC92176](https://www.ncbi.nlm.nih.gov/protein/443428676) |
| CYP82D33 | *Ocimum basilicum* | [AGF30364](https://www.ncbi.nlm.nih.gov/protein/451167578) |
| CYP82D62 | [*Mentha x piperita*](https://www.ncbi.nlm.nih.gov/Taxonomy/Browser/wwwtax.cgi?id=34256) | [AGF30366](https://www.ncbi.nlm.nih.gov/protein/451167582) |
| CYP93B23 | *Ocimum basilicum* | AGF30365 |

# Table S4. Sequences of flavonoid C-8 hydroxylase gene candidates, primer sequences and transcription units.

| Candidate flavonoids C-8 hydroxylase genes | |
| --- | --- |
| Name | Sequence |
| Rg3610short | CCCCATTTCCCCTCCCTTAGACCTTACCAGTATACAAGCTGAACTCGATATGCCCAACGCCGATTCCTCCCAGCTCGACGTCGTGGTCATCGGCGGTGAGTGAAGGCGGCGTTGTCGTTCCGCCAGCGTGCAGACAACCTTCTCACGATCTCACGCCGCAACTTGCACAGCTGGCATCGCCGGACTTGCGGCCGCGACCGCATTGCGGCACCACAACGTCACCGTCCTCGAGCAGTCTCGCTTGAAGGAAGAAGTCGGCGCCGCCATCCACCTCGGACCGAACGCCGCCAAGATCGCCCTCGGCTGGGGCATGTCGCTTGACAACCTCAACAGCCCGGAGACGCAATGGTACCACGAACTCGGTCAAGACGGCAAGACCCACTTCAAGATCCCCATTTTCGCGAGGAAGGAGTTCGGCGCGCCGTGGCTCCTGAACCACCGAGTCGACCTCCACAACGAGCTGCGTCGGCTTGCCACGACGGAAGCCGGAGAGGGCAAGCCAGCTACCGTCCGCACCGCATCGCGGGTCGTCCAGATCGACCCTGAGGCAGGTGTGGTCGAGCTCGCGGACGGCGAAAAGATCCACGCGGACGTGATCATCGCCGCCGACGGCATTCATTCGGTCGGGCGCACTGCCGTCCTCGGCCAGAAGCTCGTCGCCAACAGGTCGGGACATTCGGCCTACCGGGCTCTTATCCCGCGCGACCGTCTCCTCGACAACCCGCGCGCACTCGCTATCCTCGACGGAGATGAGAAAGGTATGGGCTTGACGACCTTTATGGGATCGGACCGGCGTCTGGTCGCCTACCCGTGCCGCAAAGGAACCTTGCTCAACATCGTCGCGATCGTGCCGGACTCGGAAGCGGAGAGCTCTACAGAGGAGTGGCACGTACAGGGAGACCCCGAGAAGCTCCTCAAGTCCTTCGAGAATTTCTGCGACGATGCCAAATTCATCCTCCGCGCCGCTCCGAGCTGCAACTTGTGGCAGCTTCGGGAACAAGACCCGCTCGAGACCTGGACCAAGGGGCGTGTCATCCTCATCGGCGATGCCGCTCACGCCATGCTTCCCCACCAAGGTCAGGGCGGCGGTCAAGCGATCGAAGACGCCGAAGCACTCGCGGTCGTGCTCCCGAACTCGACCCCTGCCTCAGCAGTACCGGAGCGTCTCCAGCTCGCCGAGAAGATTCGTTACGAACGAGCGACCCGCATCCAGTCGTACTCGCGGGAGAAGGCCCTCGGACCCAAGCCGGGCGAAAAGGTCGTCAACGCGCAGGAACACGCCGCTTACAACTTTGGGTATGCCGGCGCGCGCGACTACGCCGAGAAGCACGGGATCGCCTTGCCCGTGTCGGCCTGAGAACGTCGAGGCAAGTTTTGTCTGTCGGTTTCGTTGTATACGCAGTCCATGTAGAACTCGTATCGAGAGGTGGTTGTGAAATCAAGTTTGAAAGTAAAAAAAAAA |
| Rg3610long | CCCCATTTCCCCTCCCTTAGACCTTACCAGTATACAAGCTGAACTCGATATGCCCAACGCCGATTCCTCCCAGCTCGACGTCGTGGTCATCGGCGCTGGCATCGCCGGACTTGCGGCCGCGACCGCATTGCGGCACCACAACGTCACCGTCCTCGAGCAGTCTCGCTTGAAGGAAGAAGTCGGCGCCGCCATCCACCTCGGACCGAACGCCGCCAAGATCGCCCTCGGCTGGGGCATGTCGCTTGACAACCTCAACAGCCCGGAGACGCAATGGTACCACGAACTCGGTCAAGACGGCAAGACCCACTTCAAGATCCCCATTTTCGCGAGGAAGGAGTTCGGCGCGCCGTGGCTCCTGAACCACCGAGTCGACCTCCACAACGAGCTGCGTCGGCTTGCCACGACGGAAGCCGGAGAGGGCAAGCCAGCTACCGTCCGCACCGCATCGCGGGTCGTCCAGATCGACCCTGAGGCAGGTGTGGTCGAGCTCGCGGACGGCGAAAAGATCCACGCGGACGTGATCATCGCCGCCGACGGCATTCATTCGGTCGGGCGCACTGCCGTCCTCGGCCAGAAGCTCGTCGCCAACAGGTCGGGACATTCGGCCTACCGGGCTCTTATCCCGCGCGACCGTCTCCTCGACAACCCGCGCGCACTCGCTATCCTCGACGGAGATGAGAAAGGTATGGGCTTGACGACCTTTATGGGATCGGACCGGCGTCTGGTCGCCTACCCGTGCCGCAAAGGAACCTTGCTCAACATCGTCGCGATCGTGCCGGACTCGGAAGCGGAGAGCTCTACAGAGGAGTGGCACGTACAGGGAGACCCCGAGAAGCTCCTCAAGTCCTTCGAGAATTTCTGCGACGATGCCAAATTCATCCTCCGCGCCGCTCCGAGCTGCAACTTGTGGCAGCTTCGGGAACAAGACCCGCTCGAGACCTGGACCAAGGGGCGTGTCATCCTCATCGGCGATGCCGCTCACGCCATGCTTCCCCACCAAGGTCAGGGCGGCGGTCAAGCGATCGAAGACGCCGAAGCACTCGCGGTCGTGCTCCCGAACTCGACCCCTGCCTCAGCAGTACCGGAGCGTCTCCAGCTCGCCGAGAAGATTCGTTACGAACGAGCGACCCGCATCCAGTCGTACTCGCGGGAGAAGGCCCTCGGACCCAAGCCGGGCGAAAAGGTCGTCAACGCGCAGGAACACGCCGCTTACAACTTTGGGTATGCCGGCGCGCGCGACTACGCCGAGAAGCACGGGATCGCCTTGCCCGTGTCGGCCTGAGAACGTCGAGGCAAGTTTTGTCTGTCGGTTTCGTTGTATACGCAGTCCATGTAGAACTCGTATCGAGAGGTGGTTGTGAAATCAAGTTTGAAAGTAAAAAAAAAA |
| Rg7441 | GACCGATTGCCTCCAACGCTCGCAACGACTTCATCGATTCTGCGCCGCCAACACCTTCTGCAGGATTCGTTGATCTTGCTCGAGGGTCCCGGCTGTGCTGAGCGAGGTCGTCACACGACGTCCGACTATGCGACGAGAAGCGCGGCGCGCACCGACAGCAAGTGTCTTCGCCGGCAATAGCGGACCAGCCTCACTCGTCGTGGCGTCACGACCACCCTTTCTCCAGCACTTCATGTGCAATGCACTGTCGACGCGTCCAACACAGCCAACCAAGCGTGCAGCGCAGATCATCGGCGCTAGCGAGACCTCTTTGAAGGCGGCGCCGTCGCGGAGAGAGAGAAGCAGGATATGGCCGGACGCGCTGCGAGATGCTGCTGACGCGCGGACCATTTGCCCGTCAAGCAGATGTCCAGACACCGTGAGGCCTCCATCGTTCGAAGCTGCGAGGGTGCGTACGCAGCGTGGCCGGAAGGAAGCAGAACGGATCTCCAGCGGCAGCTCCCGCGTTGACGTAGCTGGGCGCGGGCGGCGGCGTATGCATGGCATCAAGCGTGGGAGTGCGCCGAAGGTCGCTTGCGCGGAGCATGCGAGTCTCGATTGTGTACTCGAGAGTGCAGATGCATGCCTGCAACTGGAGAGCAAGCGCGCTCAGCCGCGGCCTTCGGCCGAGAAGCGAGAAGGAGAAGATTTTCTTTGGCGCGGAAGCGGAGCAAGCACGTCTTTGACGTCGAGTTGGTTGCTATACAGCAGACTCTGTACGCTTAGCACGAGTCGCGGTGCACCTGCCAGCAGCTCAGCTGATGGTCGCGACTGCCGAGATGCCAGCCGCTGCTCTTCCTCGCGCGGTTCTCTCCAACCTCTCGGTCGGCATCGTTGGCGCAGGGATCGGCGGACTGGCAACAGCGGTCGGTCTCGCCGGGCGAGGCTTCAAGCGAGTGACCGTCTACGAATCAGCACCCGAAGTTGGCGAGGTCGGAGCAGGAATCCAAGTCGCGCCCAACTTTTGCCGCGTGCTGTCCCACTTTGGCGTCTTTGACCGGCTAAAGGCGCAGGCAGTCCGACTCGAAGGCGCAAACGTGCGGCGATACAGCGACAACGAAATCATCAACTCGACGTCGTTCGCGAGCCTGGAGAAGGAGTTTGGCGCACCGACCTATGTCGTACATCGGGCGGATCTGCATCAAGCGCTTTTGGACAGGGCACTCGAACTCGGCGCCACTCTCCAAACCAACGCACACGTCGAGAATGTCGACTTTGACAAGACGCTGCTCAAGCTGCGCGGTCAGCCCGCGCTCTCGCACGACCTCATCATCGCCGCAGACGGAATCAAGTCGGGCATTCGCTCGCAGATGATGGCGCGGAGAGGCGAAGTCGACGAGACGATCCCAACCGGCGAGGCAGCTTACCGCGTCATCTTGCCGCGGTCCGACATGGAGCAGGATCCGGAACTGAAAGCACTAATCGATGCACCTATCGCGACCCGCTGGATCGGTCCCGATGCGCATGTCGTCGCGTACCCGATCAAGGCACAGAACGCCTTCAACATCGTCACGACTCACATCAGCAACACCGTCGGCTTGACCGAGGACTGGACGGCTCGCGCTTCGAAAGACATCATGCTGAAGCGGTTCGAGGGCTGGACCGAGACGCTCCTCAAGTGCTTGCGGCTAGCCCCGCCGGGCGAACTCGTCGAATGGGCTTTGCGGATCCACCTCCCCCTCACCGGCTGGATCGACGGCAACACGGTCCTCCTCGCCGACGCCGCTCACGCCACCTTGCCGCACATCGCTCAGGGCGCCGCCCAAGCCGGTGAGGACGCCGCCGTCCTCAGCACGCTCTTGGCCAAGTGCCAGACCAAGGAGGAGGTGCCGGCTGCGTTGCGGATGTATGAGAAGCTGCGCAAACCGAGGGCGGACTGGGCTGTCGAGATGGCTCGCATCACGGGCGAGAACCTTCACATGGCGGACGGTGCTGCTCAAAAGGCGCGCGACGAGGCACTCAAGCGAGCGGCGGCGGGTGGCAAGTCGCCCGACCGCTGGGGCGACAAGGAGACGCAGCGCCGCCTCTACGGCCTCGACGTCGTCAAGCAGGCCGACCAGGAATACGCCCGCCTCTAAATCTCCTCTTTAACCCTTGACCCTCCTCTTTAGATACTACAGGTTTCCGTGTTTCGTATAGATTCTCAACGAATCGGTTGTTGTCCGGCAATTAGTCCCGCATGGAGATTCCTCTCTCCGTTCTCTTTTCTCCTGCATTTGCATATCCATCTCACTGTCTTAAAAAAAAA |
| Rg6421 | CGTAGCTAACAACTCAGAGACCGAGTCTGACAAGCTGCAGCTCTTCCTGTCCGAGCATCGGACCGGTCTCTGCTAGCTCTGAAGAACTCCACTGCGCGATCGATCGCACACGGAGACCCGCTCGGCTGTCATCATCATCGAGCGAATCCTTCTCGACAGCCACAGCTGAGCTCCGAAGCGAACACGAACCGGCAATATGAGCAAAGCGACCCGATCAGATACCACCCAGGCGGATGACCGCACGGCGCTCGTAGTCGGCGCCGGGCTGGTCGGGACCCTGTGTGCTGCCATGCTCGCCTCTCGAGGCTGGACCGTCACCCTGATCGAGTACCGCGCCGACCCTCGTCTCGGCACGCACGCCGAACGCGCCAGGTCCATCAACCTCGCCTTGTCGCCCCGAGGGATCGAGGCGATCCGGAGTGTGAGCGAAGAACTCGTCGAACGGGTCCTCAAGGAAGGGATTGAGATGAGGGGGAGGATGGTCCACAAGAAGGCCAAGAGGGAGGGCGAACCGGTCGAGAAGGACGGGCAGGACTATGGCAATTACGACGAGGGGGAATGCATCCGCTCGACGTCGAGGACTGGACTCGGGATCCAGTTGCTCGATCACTTGGACGGGTTGCCGAAACAAGGGAGGGGGAGCGTCACGACCCTCTTCGAGACGAAACTCGTCGAGATGGACTTGCGCAAGGACCACGGGGTCGACGTCGTCCTGCAGAAGAAGGGTCGGGACGGCGAGAAAGGGCACTTTGACTTTGTTGTCGGAGGTGACGGCGCGTACAGTCAAGTCCGGCAGCAGATGATGCGGGGATCTCGACTCCGCTTCGACTTTCGACAGTACTTCGCCAAGCACTCGTACCTCGAGCTGTCCATCCCGGCTGGTCCAAACAACACGTTCCTGCTCGAGCCGAATTACCTGCACATCTGGCCGCGCGGCGAGTTCATGCTGATCGCGCTGGCAAACCTGGACAAGTCCTTCACGTTGACCCTCTTTGCGCACGACTCGACCTTTTCGTCGATCGATGCTCAACTCGCATCGTCCGACTCGTCCAAGTCGAACGGATCGAATCCGGTTGTCGAGCTGTTCCGAAAAGAGTTCCCGGACGCGCTCGAACACATGGGCGAGGAAGCACTCTTGCGGAGCTGGAAGGAGAACCCGAAGGATGGGTTGATCACCGTCGAGTGCTCGCCGTACCACTACCAAGACAAGGTCTTGCTCATCGGCGACGCCGCCCACGGCATGGTCCCCTTCTACGGTCAAGGCATGAACTGCGGCTTCGAGGACGTCCGCGTCCTGTCGTCGATGCTCGACCACTTTGGCGCCTCGCCGTCGTCTTTGGTGCCGTCACCTTTGCCCTACTCGGAGGTGACGCCGCCGCTTCCGGTACCCATGGACCCGTTATCGAAGGAGCCGTTGGTGGGCAAGACCCCGCTCGCGCAGGCCCTCGCGGCCTACACGACGATCCGGGCGCCGTCTCTCTCGGCTATCCAGCAGCTCGCCCAGAGGAATTATACCGAGATGGCGTCTTCAGTTCTCTCGCCGCTGTACCTCCTCCGAAGAGGGCTCGACTCGCTCTTGTCTTCCCTCTCGACGCTGCCGCCGTTTGCGATCCCGCGGGATCAGGACCCGACCAAGGACCGCGGCGGTCGATGGGAGAGCCTGTACCGGATGGTGACGTTCCGGCCTGGACTCGCGTACGAGGAAGTGATCCGGAGGAGCGAGTGGCAGCAGCGGGTGCTGCGGCGAGCGGTGCAGGCCATCGGAGGAGCCGTTTTCGTTGGAATGGCGGTCGGCGTTGGACTCAAGTACGCGGGGCGGTATCGTCTAGTCAAAGTAGCATAGCCATCCCCCTGAATGTAACGACTCGTTGCACTAAAAAAAAAAAAAA |
| Rg2726 | CCCAACCGCCCCAGCTTGGTGAAGCTCGAGCTCATCCTCGCTACAGGGCAACAAGAGGGCAAGTCGAGTCTCCAGCCGCAGCGTGAGGTCTTGCGCGGATCTGCGCCGGGCGTCGGTCCTTTCCCGATTCTCGCACCTTCGCGTTTCTCGCCGGCTCCCGCTTGCTCGCCTCGCTGTCCCGACACGCTGACGATCCCTCCCAGACTCGGACTTGAGCTTGCTTACAAGGTCAGCCTACGTCGCTGCGACGGATGGCGCCCCGCATGCTTGCGTTTGCGCGCTCGGCTCATCAGCCATATGCCTTGGCAAAGACGTGACTTGATTCCGTGGCTGTCTCCTCTCATTCGCTGCTTGCTTTGGACTCGCGAGTAAAGCACGGATGCAGCTGCTCAACCTCTGCCCGCCCTTCTTGCGCCGAGGAGTGAAGAAGAAGAGCTCTACGCCGAAAAGTCACCGCACTCCCGCTCAACCGACAGCGACGAGTTCTACCTCGGACGCGGCGAGTCCTGTAAACTCGCCACGAGATTCGCATGAACGACTGGTCAAGGGCAAGCCTCCACAGAAGGAAACCCGAGTCAAGTCTGGAACGTCAGGAGGGAAACTGGTTCGCCTGCCACCGAGCGAGTATACGGAGCCACCATCGCAAGTGTTCCCTTGCGCCCCTTGCGCAGAAAGCCTTGGCGACGGCGATACGTTTGCATTCGACGACCTCGCTCGCAACGAAAATCTGCGGGTCGTAATCGTAGGAACGGGATTCGCCGGATTGGCGGCCGCTATCGCCTGCGCTCGACAAGGGTTCTCCGTCACAGTGTGCGAGCGGTCTGCCGGCATCTCGCCGCATGGCGACTCGATCCTCTTCGGAGCAAACGCCAGTCGGATCTTTTACCGGTGGGGCATTGGAGGAGACCTGTATCGGAGGGGAGGGAGTAGAGGGGGACGGTGGATCTTTCAGGATGAAAAGGGCAAGATAGTTGAGCAGGTTCAGATCGGTAACATGATCGATCAACACGGCGCGCCGATTCTGCAAGGTCGACGGTCAACTTTTCTCGGCTCGCTCGGTACCGAGGCTCGCCTGCTCGGAGTCACGATTCGACTCGCAAGCGAAGTCGTCAAATACTGGGACTCGAACGAAGAGCCGGCGGTGGTCTTGCGAGGGGGAGAGACTTTGCGAGCGGATGTCGTCATTGTGGCGGATGGTGTGCACTCTCCGGCGCGCTGGCTACTCACGCCGCACGCTCGCAAGGCCGCCGAAAAAATCCCTTCTGGCTATTCGGTTCATCGTTCCGCCATCTCGGCGGAAAAGCTCAAGGCTGATAGCCGCTGTTCGCATCTGCTCGACGGGACCATACGCACCTGGCTCGGCCCCGATGCACACGCTTGTACATATCCACTCGACAACGGCAACGGTCTTGCCTTTACCTACACGCACCGCGATGACGGAAGCCTCGCCTCGCTCGACTGGCGCGACAAGAAGCCGATCGCAGGCATGCTCAAGGAACTGCAAGGATGGGACCCCGTCCTCTTGGCTGCCCTCTCGCACTTTCCGAGCTCGCTGCATTGGACTATCGTTGACGAGAAGCCGGAGGAGGAATGGATCAGCGCTGGAGGGCGAATCTGCTTCGTCGGCGACTCTGTCCACCCTATGATGCCGACAGCTTTCCAAGGTGGCTCACAAGCGATTGAAGATGCAGCAGCGATAGCTCTTTGCTTGGCTCTCACTGGTGGACGTCCCGATGGTGTGCCGTTAGCACTCGAGACGTACGAAGCGCTGCGACGACCGCGCATCAAAGAGGCACAGGCGCTGGGAAAATTGCAACAAGATCTGTGGCATCAGTATCTCCCACCGCCCGCCGCTCGCCGCCGCAGGCTTTCCCGCTCGTCGGATTCTTCAACAAATGCGCCCGGTCCAAGTCGACCCTCACTTTGCCCGCTTACCTTCGCCCTGTACGACTACGACGTGGAGAACTATGTTCTCGAGCACTTCGAGACGTTTGCGCGCGCGATCGATCCCGACTTTGTGCTCAAGACGAGCTGGCGGGAGGATGCGGCTCGCAAAGCCAACATCGCGCTCGACGTGAGGCGGGCTTCCTGGGCCGACTCGATCGAAAGGGAGGGGATGAAAGTTGATGAGGTCGGATGACGGTCGCTGTTTAGATCTCAAGGAGTCCGACAGATGACCGGACTCGTTGAAATCGCAAAACTTGAAACGTGAA |

| Primers | |
| --- | --- |
| Name | Sequence |
| R24_SEVA182 | AGCGGATAACAATTTCACACAGGA |
| F24_SEVA182 | CGCCAGGGTTTTCCCAGTCACGAC |
| rhaB_expression module_Fwd | GGCGCTTTTTAGACTGGTCG |
| rha S middle F | GGACGGGATGGCTTTCTGCAATAA |

| Transcription units | | | |
| --- | --- | --- | --- |
| Plasmid | Characteristics | Strain | Used for |
| Lv1 Rg3610short | Km^R^, pBBR1, Lv1 pSEVA23g19g1 vector, pJ23100, RBS T7 from pET28, Rg3610short gene, and T7 terminator from pET28 | DH5α | *In vivo* assay |
| Lv1 Rg3610long | Km^R^, pBBR1, Lv1 pSEVA23g19g1 vector, pJ23100, RBS T7 from pET28, Rg3610long gene, and T7 terminator from pET28 | DH5α | *In vivo* assay |
| Lv1 Rg7441 | Km^R^, pBBR1, Lv1 pSEVA23g19g1 vector, pJ23100, RBS T7 from pET28, Rg7441 gene, and T7 terminator from pET28 | DH5α | *In vivo* assay |
| Lv1 Rg6421 | Km^R^, pBBR1, Lv1 pSEVA23g19g1 vector, pJ23100, RBS T7 from pET28, Rg6421 gene, and T7 terminator from pET28 | DH5α | *In vivo* assay |
| Lv1 Rg2726 | Km^R^, pBBR1, Lv1 pSEVA23g19g1 vector, pJ23100, RBS T7 from pET28, Rg2726 gene, and T7 terminator from pET28 | DH5α | *In vivo* assay |
| Lv1 N-His-Rg3610short | Km^R^, pBBR1, Lv1 pSEVA23g19g1 vector containing RhaS gene, RhaBAD promotor, RBS T7 from pET28, Rg3610short gene fussion with N-terminal His tag, and T7 terminator from pET28 | DH5α | Overexpression analysis |
| Lv1 N-His-Rg3610long | Km^R^, pBBR1, Lv1 pSEVA23g19g1 vector containing RhaS gene, RhaBAD promotor, RBS T7 from pET28, Rg3610long gene fussion with N-terminal His tag, and T7 terminator from pET28 | DH5α | Overexpression analysis |
| Lv1 N-His-Rg7441 | Km^R^, pBBR1, Lv1 pSEVA23g19g1 vector containing RhaS gene, RhaBAD promotor, RBS T7 from pET28, Rg7441 gene fussion with N-terminal His tag, and T7 terminator from pET28 | DH5α | Overexpression analysis |
| Lv1 N-His-Rg6421 | Km^R^, pBBR1, Lv1 pSEVA23g19g1 vector containing RhaS gene, RhaBAD promotor, RBS T7 from pET28, Rg6421 gene fussion with N-terminal His tag, and T7 terminator from pET28 | DH5α | Overexpression analysis |
| Lv1 N-His-Rg2726 | Km^R^, pBBR1, Lv1 pSEVA23g19g1 vector containing RhaS gene, RhaBAD promotor, RBS T7 from pET28, Rg2726 gene fussion with N-terminal His tag, and T7 terminator from pET28 | DH5α | Overexpression analysis |
| Lv1 N-His-Rg3610long_BL21 | Km^R^, pBBR1, Lv1 pSEVA23g19g1 vector containing RhaS gene, RhaBAD promotor, RBS T7 from pET28, Rg3610long gene fussion with N-terminal His tag, and T7 terminator from pET28 | BL21 (DE3) | Biochemical characterization and substrate specificity |
| Lv1 N-His-Bm_GDH | Km^R^, pBBR1, Lv1 pSEVA23g19g1 vector containing RhaS gene, RhaBAD promotor, RBS T7 from pET28, Bm_GDH gene fussion with N-terminal His tag, and T7 terminator from pET28 | BL21 (DE3) | Regeneration system |

# Figure S1. Nucleotide sequences encoding the amino acids fdeE - line 1, and Rg3610long (RgF8H) - line 2. Rg6421, Rg2726. Highly conserved FAD and GD motifs were highlighted in boxes using red and blue underscores, respectively.


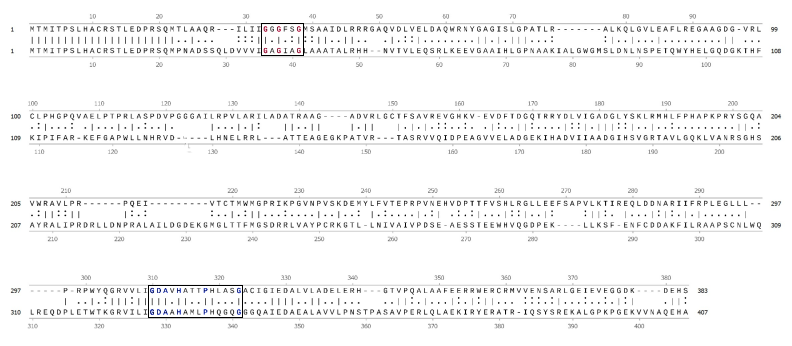


# Figure S2. LC-MS analysis of naringenin hydroxylation by RgF8H. (A) Mass spectrum of naringenin, (B) 8-hydroxynaringenin.

A

B

# Figure S3. LC-MS analysis of eriodictyol hydroxylation by RgF8H. (A) Mass spectrum of eriodictyol, (B) 8-hydroxyeriodictyol.

B

A

# Figure S4. LC-MS analysis of pinocembrin hydroxylation by RgF8H. (A) Mass spectrum of pinocembrin, (B) 8-hydroxypinocembrin.

A

B

# Figure S5. LC-MS analysis of hesperetin hydroxylation by RgF8H. (A) Mass spectrum of hesperetin, (B) 8-hydroxyhesperetin.

A

B

# Figure S6. LC-MS analysis of luteolin hydroxylation by RgF8H. (A) Mass spectrum of luteolin (B) 8-hydroxyluteolin.

B

A

# Figure S7. LC-MS analysis of apigenin hydroxylation by RgF8H. (A) Mass spectrum of apigenin, (B) 8-hydroxyapigenin.

B

A

# Figure S8. LC-MS analysis of chrysin hydroxylation by RgF8H. (A) Mass spectrum of chrysin, (B) 8-hydroxychrysin.

A

B

# Figure S9. LC-MS analysis of diosmetin hydroxylation by RgF8H. (A) Mass spectrum of diosmetin, (B) 8-hydroxydiosmetin.

#

A

B

# Figure S10. LC-MS analysis of 7,4’-dihydroxyflavone hydroxylation by RgF8H. (A) Mass spectrum of 7,4’-dihydroxyflavone, (B) 7,8,4’-trihydroxyflavone.

B

A
